# Chlorophyll-*a* unveiled: unlocking reservoir insights through remote sensing in a subtropical reservoir

**DOI:** 10.1007/s10661-024-12554-w

**Published:** 2024-03-27

**Authors:** Kudzai S. Mpakairi, Faith F. Muthivhi, Farai Dondofema, Linton F. Munyai, Tatenda Dalu

**Affiliations:** 1 Department of Earth Sciences, Institute of Water Studies, University of the Western Cape, Bellville, 7535 South Africa; 2 School of Wildlife Conservation, African Leadership University, Kigali, Rwanda; 3 Department of Geography and Environmental Sciences, University of Venda, Thohoyandou, 0950 South Africa; 4 Aquatic Systems Research Group, School of Biology and Environmental Sciences, University of Mpumalanga, Nelspruit, 1200 South Africa; 5 South African Institute for Aquatic Biodiversity, Makhanda, 6140 South Africa; 6 Stellenbosch Institute for Advanced Study, Wallenberg Research Centre at Stellenbosch University, Stellenbosch, 7600 South Africa

**Keywords:** Water resource management, Chlorophyll-*a*, Nandoni Reservoir, Water quality, Spectral bands, Spectral indices

## Abstract

Effective water resources management and monitoring are essential amid increasing challenges posed by population growth, industrialization, urbanization, and climate change. Earth observation techniques offer promising opportunities to enhance water resources management and support informed decision-making. This study utilizes Landsat-8 OLI and Sentinel-2 MSI satellite data to estimate chlorophyl-*a* (chl-*a*) concentrations in the Nandoni reservoir, Thohoyandou, South Africa. The study estimated chl-*a* concentrations using random forest models with spectral bands only, spectral indices only (blue difference absorption (BDA), fluorescence line height in the violet region (FLH_violet), and normalized difference chlorophyll index (NDCI)), and combined spectral bands and spectral indices. The results showed that the models using spectral bands from both Landsat-8 OLI and Sentinel-2 MSI performed comparably. The model using Sentinel-2 MSI had a higher accuracy of estimating chl-*a* when spectral bands alone were used. Sentinel-2 MSI’s additional red-edge spectral bands provided a notable advantage in capturing subtle variations in chl-a concentrations. Lastly, the *–*chl-*a* concentration was higher at the edges of the Nandoni reservoir and closer to the reservoir wall. The findings of this study are crucial for improving the management of water reservoirs, enabling proactive decision-making, and supporting sustainable water resource management practices. Ultimately, this research contributes to the broader understanding of the application of earth observation techniques for water resources management, providing valuable information for policymakers and water authorities.

## Introduction

Water resource management is crucial in sustaining human populations, ecosystems, and economic activities globally (Chawla et al., 2020; Sheffield et al., 2018). Many regions worldwide are experiencing water stress, and scarcity issues (Bhattacharya & Raghuvanshi, 2018; Leal Filho et al., 2022). Population growth, industrialization, urbanization, and climate change are reasons behind these issues and have affected water availability and quality globally (Hanjra & Qureshi, 2010; Meyer & Turner, 1992). In addition, ineffective water management practices, inefficient distribution systems, and over-extraction from rivers and aquifers have also exacerbated the problem (Nagara et al., 2015). Earth observation techniques have the potential to lessen these challenges by providing valuable data on reservoir water quality, allowing for proactive planning and management, of water resources (Matthews, 2014; Matthews & Bernard, 2015).

Adopting earth observation technologies to monitor reservoirs has brought about a pivotal advancement in assessing water resources management (Bangira et al., 2019; Matthews, 2014). One commonly used parameter to assess water quality with earth observation technologies is chlorophyll-*a* (chl-*a*) concentration (Kravitz et al., 2020; Matthews, 2014; Matthews & Bernard, 2015). Chlorophyll-*a* (chl-*a*) presence is an important indicator of water quality and a crucial factor in understanding the dynamics of algal blooms (Kravitz et al., 2020). This dual role provides valuable insights into the ecological health of reservoirs. Algal blooms are usually a result of excess nutrients in water resources and can thrive when chl-a levels are elevated (Liao et al., 2021; Summers & Ryder, 2023). These alga blooms can lead to the proliferation of harmful algal species that produce cyanotoxins (e.g., microcystins) detrimental to human health and aquatic life (Matthews et al., 2010). Ingesting water contaminated with these toxins can lead to gastrointestinal issues and more severe conditions such as liver damage and neurological disorders (Diez-Quijada et al., 2021; Flores et al., 2018). In aquatic life, these toxins can deplete oxygen levels in water bodies, lead to the death of fish, and disrupt the balance of aquatic ecosystems (Flores et al., 2018). Earth observation techniques can effectively monitor these alga blooms through assessing chl-*a* levels and give insights on areas with potential algal blooms (Dzurume et al., 2022; Malahlela et al., 2018). By closely monitoring chl-a concentrations and their relationship to algal blooms, we can not only ensure the ecological balance of reservoirs but also safeguard the well-being of the aquatic life and human populations.

Medium-resolution satellite sensors such as Landsat-8 Operational Land Imager (OLI) and Sentinel-2 Multispectral Instrument (MSI) have been effectively utilized for water resources monitoring in most environments (Dzurume et al., 2022; Malahlela et al., 2018). Both satellites have multispectral sensors that capture data across different spectral regions. These spectral regions are valuable for assessing various aspects of water resources, including chl-*a* concentrations (Barraza-Moraga et al., 2022), turbidity (Magrì et al., 2023), and suspended sediment loads (Zhang et al., 2022a, 2022b). The temporal scale and historical images available from these sensors allow for adaptive management strategies and timely assessments of water quality (Matthews et al., 2010; Smith & Bernard, 2020). This allows for cost-effective water management practices at different spatial scale from the synoptic view offered by Sentinel-2 MSI and Landsat-8 OLI (Matthews et al., 2010).

South Africa is a water-scarce country with highly variable rainfall patterns. The National Water Act (Act 36 of 1998) provides a legal framework for water resource management in South Africa, including allocating and protecting water resources. Despite this legislative framework, South Africa still needs to overcome several challenges to effectively manage its water resources. These challenges include but are not limited to (1) competing demands for water use, (2) limited financial resources for managing water resources, and (3) institutional constraints within government departments (Plessis, 2023; Sorensen, 2017). Owing to these challenges, water resources in South Africa are experiencing eutrophication, sedimentation, and deteriorating water quality (Harding, 2015; Matthews & Bernard, 2015). Governments, water authorities, and communities in South Africa can utilize remote sensing products and tools to enhance their understanding of reservoir water quality necessary in supporting evidence-based decision-making, and fostering sustainable water management practices.

To effectively monitor these threats this study proposes a cost-effective method using earth observation tools to provide essential insights into the water quality monitoring for the Nandoni Reservoir. The water quality in Nandoni Reservoir, South Africa, is under threat due to various anthropogenic activities, including partially treated sewage plant discharges, the introduction of harmful by-products, and inadequate water management practices (Gumbo et al., 2016; Takalani, 2022). This study assesses the use of using earth observation in monitoring water quality based on chl-a concentrations in the Nandoni Reservoir. This is unique to the Nandoni reservoir and can enhance timely water resources monitoring. Specifically, this study evaluates the utility of spectral indices only, spectral bands only, and a combination of spectral bands and spectral indices derived from Landsat-8 OLI and Sentinel-2 MSI to monitor the chl-a concentration in the Nandoni Reservoir. This research aims to contribute to understanding remote sensing techniques for water resources management and their application in South Africa’s context and facilitate informed decision-making processes necessary for water resource management. This research adds to the body of knowledge on the application of remote sensing in monitoring and managing water resources in regions impacted by similar anthropogenic activities. The findings and methodologies presented in this study can potentially be extrapolated and applied to other regions facing comparable water quality challenges, thus broadening the applicability and relevance of remote sensing techniques in addressing global water resource management issues.

## Materials and methods

### Study area

**Fig. 1:**
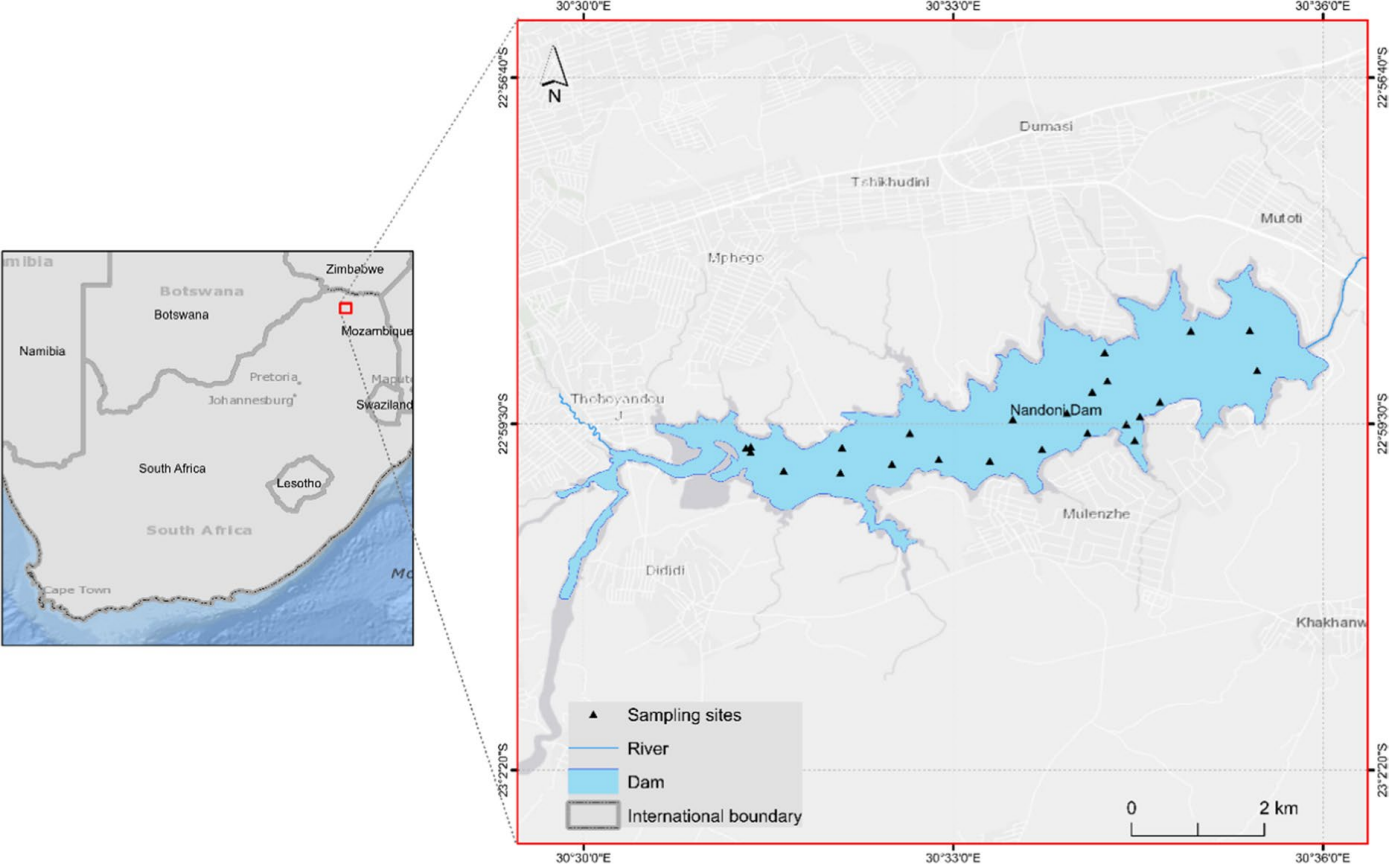
The location of the Nandoni Reservoir in Limpopo province, South Africa

The study was conducted in the Nandoni Reservoir in the Limpopo province of South Africa (Fig. 1). This reservoir, constructed between 1998 and 2005, is an earth-filled concrete structure that relies primarily on the Luvuvhu River. Covering an approximate catchment area of 1380 square kilometers, the reservoir spans a total length of 2215 m and boasts a total capacity of 16.4 million cubic meters (Gumbo et al., 2016; Mbedzi et al., 2020; Takalani, 2022). Annual precipitation within the catchment area typically ranges from 610 to 800 mm, resulting in a mean annual runoff of 519 million cubic meters (Makherana et al., 2022; Mbedzi et al., 2020). The reservoir’s topography features low-lying, undulating terrain, underlain by a gneiss sequence of the Soutpansberg group.

The Nandoni Reservoir is a crucial water resource, serving the needs of 1.3 million people in the Vhembe and parts of Mopani districts, Limpopo province (Dalu et al., 2019; Gumbo et al., 2016). The reservoir supplies water to several places and was constructed to upgrade water resource management and promote economic development through water-based recreation and tourism (Sinthumule, 2021). Specifically, it plays a significant role in supporting the communities’ water requirements and presents recreational opportunities that can foster socioeconomic development, especially for waterfront villages facing challenges related to poor service delivery and high unemployment (Gumbo et al., 2016).

### Water sample collection and processing

To determine the chl-*a* concentration of the Nandoni Reservoir, a boat was used to collect water samples at 26 randomly selected sites. These sites were distributed along the entire length of the reservoir as shown in Fig. 1. The water sample were collected from the 3rd to the 5th of December 2020 under clear skies (between 09:00 and 13:00) so that diurnal changes in water quality can be controlled.

The water samples collected to determine chl-*a* concentration was done using 1L Consol glass bottles. Integrated water samples (*n* = 2 per site) from the reservoir were taken below the surface, by directly holding the bottle into the water at a depth of 30 cm below the surface. Extreme care with the water sample bottles were taken to ensure that no air bubble was left inside. All bottles were marked with the site number after the collection, and the samples were stored in a cooler box on ice before being taken to the laboratory for further analysis.

Chlorophyll-*a* measurements in the laboratory were done to give a proxy of the phytoplankton biomass present in the water. In the laboratory, 250 mL of sampled water were filtered through 0.07 µm (i.e., diameter 47 mm) glass fiber filter (GF/F). Each filter paper was folded into half and wrapped in aluminum foil and placed them in the freezer, to protect the extracts from the light before chl-*a* extraction. Later on, the individual labelled filters were added in 10 mL of 90% acetone in 15-mL centrifuge tubes to extract the total chl-*a* over 24 h at –20 °C in the freezer as described in Dalu et al. (2013). After 24 h, the extract was centrifuged at 3000 rpm for 5 min to remove any materials in suspension. Chlorophyll-*a* concentration was determined by using spectrophotometry method which involved measuring absorbance at wavelengths of 665 nm and 750 nm using a SPECTROstar NANO (BMG Labtech GmbH, Ortenberg), before calculating chl-*a* concentration according to Almomani and Örmeci (2018).

### Satellite image processing

In this study, data from two satellite sources, Sentinel-2 MSI and Landsat-8 OLI, covering the Nandoni Reservoir were used. These images were retrieved from Google Earth Engine (GEE) and their acquisition dates (3–5 December 2020) coincided with when the point data for chl-*a* was collected. The images were retrieved as surface reflectance from GEE platform and were already processed for atmospheric attenuations and topographic effects (Mpakairi et al., 2022a, 2022b). To ensure high data quality, cloud-quality filters and a cloud mask were also applied, eliminating potentially cloud-covered or low-quality data and ensuring that only high-quality and cloud-free pixels were used for analysis (Sharifi et al., 2022).

#### Spectral indices for estimating chl-a concentration

From the retrieved satellite data, three spectral indices were calculated for each satellite sensor. These spectral indices were the blue difference absorption (BDA), fluorescence line height in the violet region (FLH_violet), and normalized difference chlorophyll index (NDCI). These indices have been widely used in previous studies for estimating chl-*a* concentrations using remote sensing data hence their use in this study (Buma & Lee, 2020; Mishra & Mishra, 2012).

The three-band algorithms (BDA) index measures the difference in absorption between the blue and green regions of the electromagnetic spectrum (Dimapilis, 2021; Gitelson et al., 2003; Johansen et al., 2018). As chl-*a* absorbs light in the blue region and reflects light in the green region, the BDA index allows us to estimate chl-*a* concentrations in water bodies by quantifying this differential absorption pattern (Johansen et al., 2018). The BDA is calculated using Eq. 1 in Table 1.

**Table 1:** The spectral indices that were used in estimating chl-a concentration

Equation number	Spectral indices name	Formulae	Source
Equation 1	Three-band algorithms (BDA) index	Sentinel-2 MSI $\frac{1}{B4}-(\frac{1}{B5}*B8b)$	(Buma & Lee, 2020)
		Landsat-8 OLI (Blue) − (Red)/(Green)	
Equation 2	Fluorescence line height (FLH) index	Sentinel-2 MSI (band3) − [(band4) + (band2) − (band4)]	(Buma & Lee, 2020; Johansen et al., 2018)
		Landsat-8 OLI (NIR band is far from chlorophyll-a peak)$\frac{NIR-Red}{NIR+Red}$	
Equation 3	Normalized difference chlorophyll index (NDCI)	Sentinel-2 MSI $\frac{Red edge-Red}{Red edge+Red}$	(Johansen et al., 2018)
		Landsat-8 OLI $\frac{NIR-Red}{NIR+Red}$	

The fluorescence line height (FLH) index is specifically designed to detect chlorophyll fluorescence, which is directly related to the concentration of chl-*a* in the water (Zhao et al., 2010). By measuring the height of the fluorescence peak in the violet region of the spectrum, the FLH_violet index provides valuable information for accurate chl-*a* estimations(Beck et al., 2016; Johansen et al., 2018). The FLH_violet can be calculated using Eq. 2 in Table 1.

The normalized difference chlorophyll index (NDCI) is a widely used spectral index for chl-*a* estimation (Mishra & Mishra, 2012). It capitalizes on the principle that chl-*a* absorbs more light in the red region of the spectrum and reflects more in the near-infrared region. By taking the normalized difference between these two bands, the NDCI index enhances sensitivity to chlorophyll-a content, enabling precise estimations of chl-*a* concentrations in water bodies. The NDCI can be calculated using Eq. 3 in Table 1.

### Estimating chlorophyll-a with random forest and remote sensing data

To estimate chl-*a* concentrations in the Nandoni Reservoir, the study used the random forest algorithm. The random forest algorithm was purposively selected because it is a reliable and highly predictive classifier capable of dealing with non-linear data (Mpakairi & Muvengwi, 2019; Mpakairi et al., 2022b). It has also been observed to outperform other classifiers such as support vector machines and Naïve Bayes (Gxokwe et al., 2022).

The random forest model requires model calibration based on a set of hyperparameters. These hyperparameters include the number of trees (*n* = 500), maximum tree depth (10), minimum samples per leaf (2), minimum samples per split (5), and random seed (50). These hyperparameters collectively influence model performance and robustness, necessitating careful calibration to optimize results (Mpakairi & Muvengwi, 2019; Mpakairi et al., 2022b).

The random forest models used point data collected in the field as well as spectral bands and indices derived from Sentinel-2 and Landsat-8 satellite imagery. Specifically, six random forest models were used to estimate chl-*a* concentrations in the study area. The first pair of random forest models were built individually using spectral bands from Sentinel-2 MSI and Landsat-8 OLI only. The second pair of the random forest models were also built using spectral indices calculated in Table 1 for Sentinel-2 MSI and Landsat 8-OLI. Lastly, the third pair of the random forest models were built using combined spectral bands and indices for each satellite sensor. The spectral bands-only models were used to assess the relationship between the spectral band values and chl-*a* concentrations, whereas the combined spectral bands and indices model were used to assess the performance of the model when provided with additional information from the spectral indices. Lastly, the spectral indices alone models were used to determine if the spectral indices alone, specifically designed to estimate chl-*a*, could independently provide accurate predictions.

### Model evaluation

To evaluate the accuracy and reliability of the random forest models for estimating chl-*a* concentrations, internal cross-validation was used considering the limited number of data points. To maximize data usage, we divided the chl-*a* measurements into a predefined number of subsets or folds (*K* = 10). Each fold was alternately used as the test set, while the remaining folds served as the training set during successive model iterations. The final model was determined by identifying the model with the highest cross-validated coefficient of determination (*R*^2^_cv_). Other validation measures there were also calculated include the root mean square error (RMSE) and mean absolute error (MAE). Internal cross-validation technique helps mitigate the risk of overfitting and provides greater confidence in the model’s ability to generalize to new, unseen data despite the small dataset (Vabalas et al., 2019). The insights gained from this evaluation are critical for validating the applicability of the spectral band and indices used and the overall accuracy of the models.

## Results

### Model performance

**Fig. 2:**
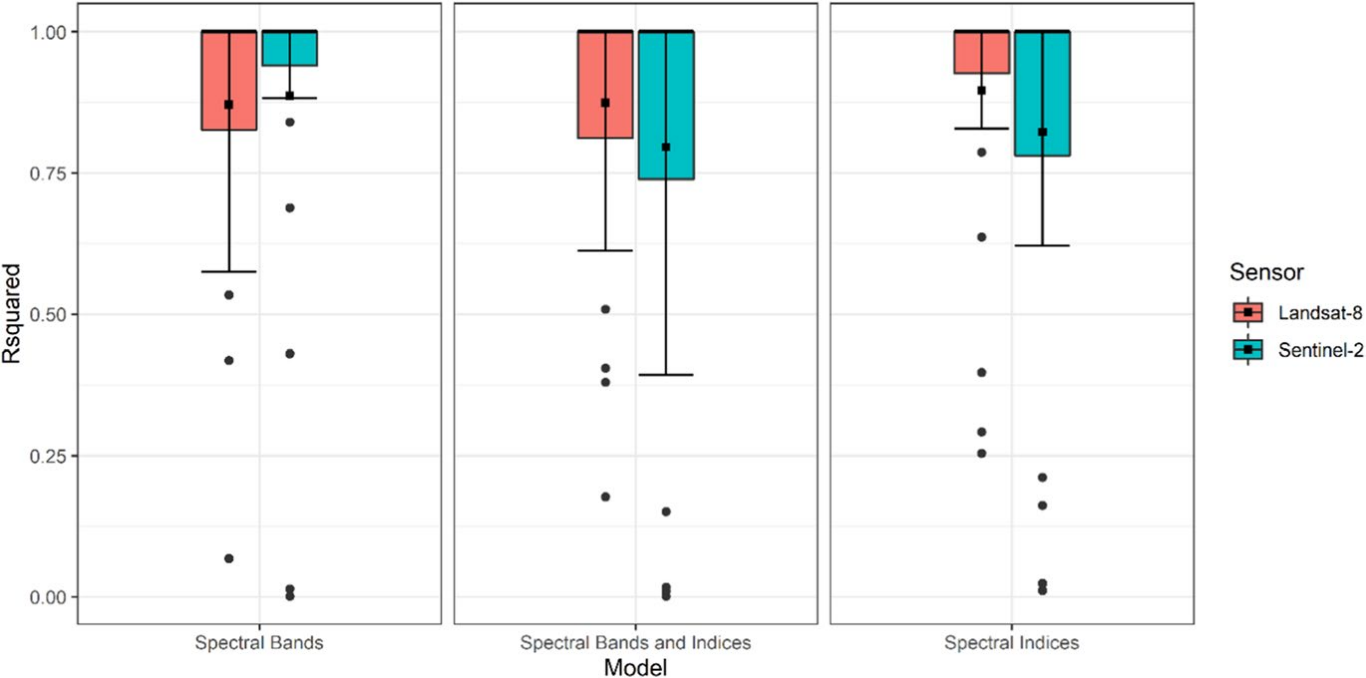
The mean difference of all models was evaluated using the cross-validated coefficient of determination (*R*^*2*^_cv_). The models were run using either Landsat-8 OLI or Sentinel-2 MSI imagery

All the models used in this study were able to estimate chl-*a* in the Nandoni Reservoir (*R*^*2*^ > 0.80) (Table 2). The spectral bands-only model performed better using Sentinel-2 MSI (*R*^*2*^_*cv*_ = 0.89) than when the model used Landsat-8 OLI data (*R*^*2*^_*cv*_ = 0.87). Using Landsat-8 OLI derived data improved the performance of the spectral indices model (*R*^*2*^_*cv*_ = 0.90) and spectral bands and indices model (*R*^*2*^_*cv*_ = 0.87). Although the predictive accuracy of these models differed, there was no significant difference (*p* > 0.55) between all the models used in this study (Fig. 2).

**Table 2:** Results for the cross-validated coefficient of determination (R_cv_^2^), root mean square error (RMSE), and mean absolute error (MAE) to evaluate model performance for all the models executed using Landsat-8 OLI or Sentinel-2 MSI data

Model	Landsat-8			Sentinel-2		
	R_cv_^2^	RMSE_cv_	MAE_cv_	R_cv_^2^	RMSE_cv_	MAE_cv_
Spectral bands	0.87	20.27	0.18	0.89	10.18	0.11
Spectral indices	0.90	30.01	0.19	0.82	50.09	0.29
Spectral bands and indices	0.87	22.30	0.06	0.80	23.53	0.03

### Distribution of chlorophyll-a concentrations

**Fig. 3:**
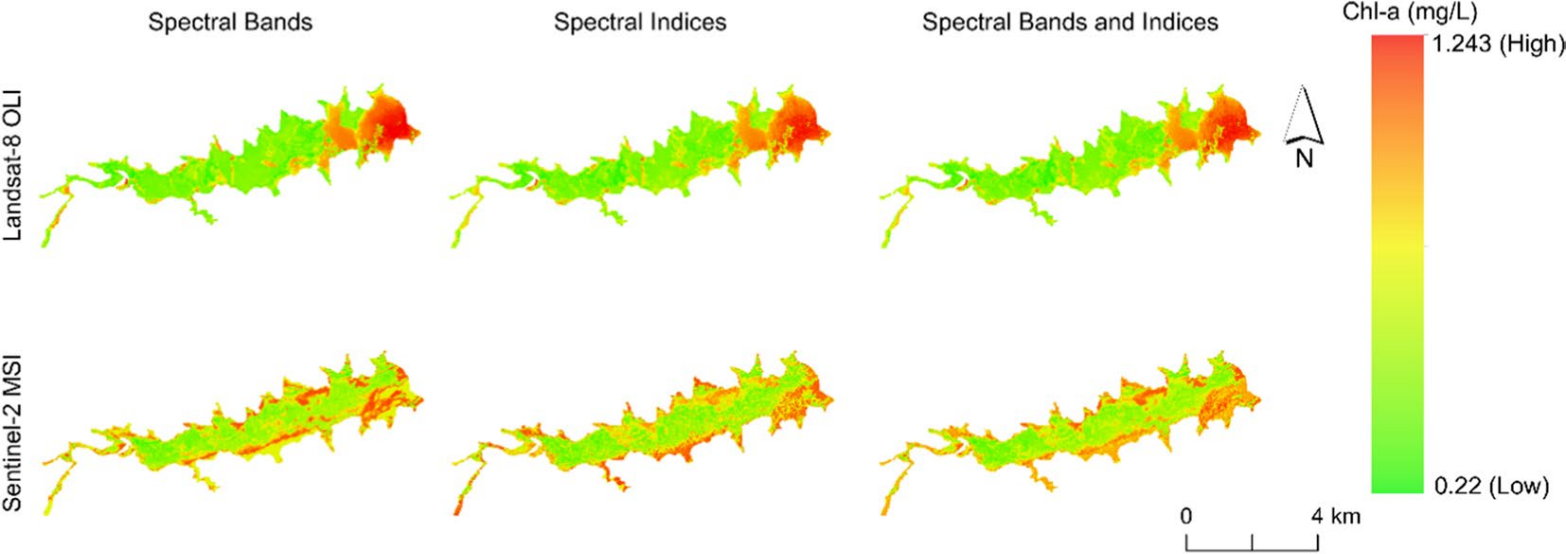
Distribution of chl-a in Nandoni Reservoir using six models based on either Landsat-8 OLI or Sentinel-2 MSI derived data

The concentration of chl-*a* differed across the reservoir depending on the sensor used. However, the concentration did not vary with the model used. Models using Sentinel-2 MSI showed that chl-*a* was high along the shoreline, and along most distributaries of the reservoir (Fig. 3). Models using Landsat-8 OLI showed that chl-*a* was mainly high closer to the reservoir wall. For both sensors and with all models, the central part of the Nandoni Reservoir had low chl-*a* concentration.

### Variable contribution to estimating chl-a concentration

#### Spectral bands model

**Fig. 4:**
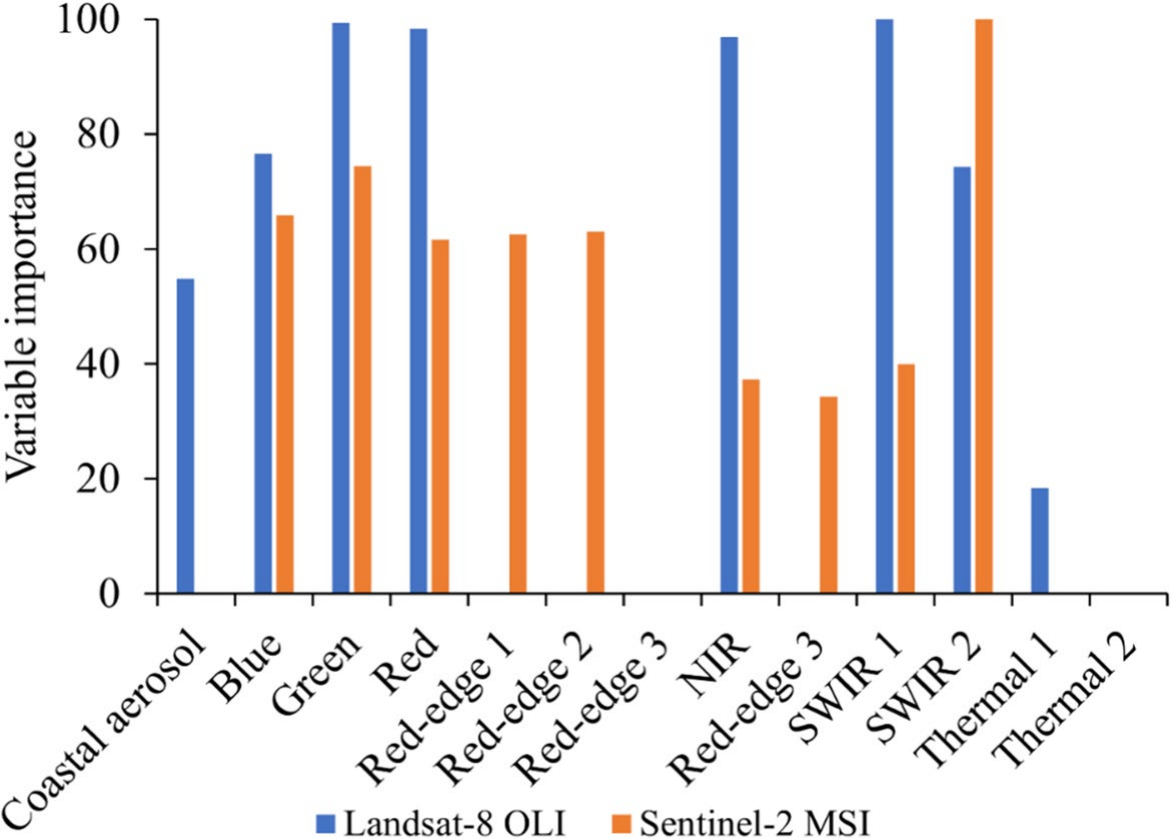
Variable contribution of Landsat-8 OLI or Sentinel-2 MSI spectral bands to the performance of the spectral bands-only model

The results showed that for the model using spectral bands only from Landsat-8 OLI data, the near-infrared, shortwave infrared, and green spectral bands contributed most to the overall performance of the model (Fig. 4). In addition, the thermal band from Landsat–8 OLI contributed the least to the overall performance of the spectral bands-only model using Landsat-8 OLI data. On the other hand, SWIR 2 and the green spectral bands contributed most to the overall performance of the spectral bands-only model using Sentinel-2 MSI data, and the red-edge spectral bands (red-edge three and red-edge four) contributed least to the same model.

#### Spectral indices model

**Fig. 5:**
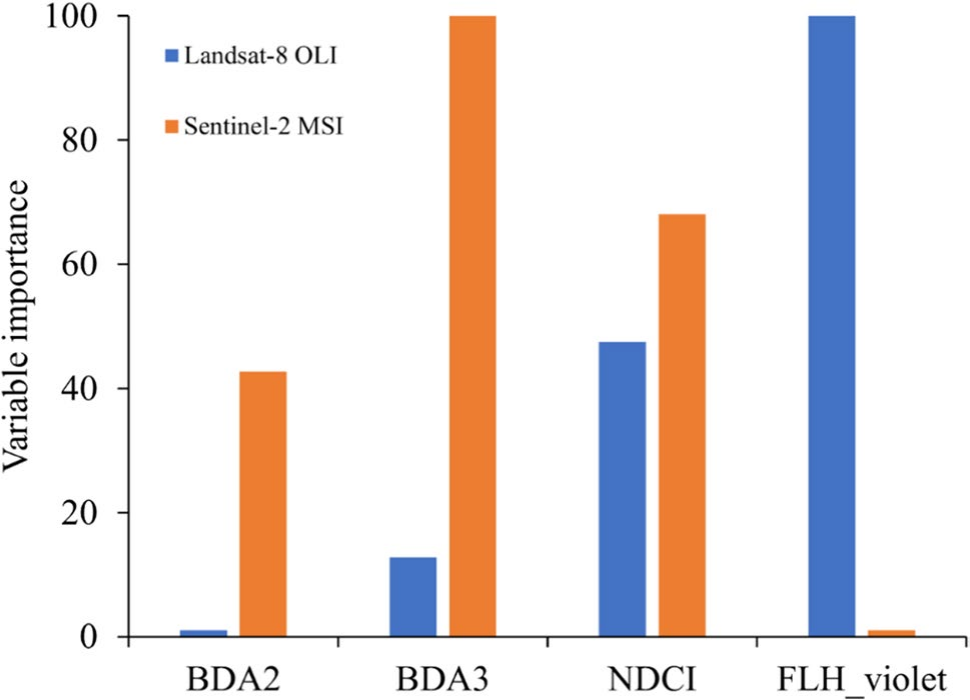
Variable contribution of Landsat-8 OLI or Sentinel-2 MSI-derived spectral indices to the performance of the spectral indices-only model

The results also showed that the BDA3 spectral index contributed most to the spectral indices model using Sentinel-2 MSI data and the FLH_violet spectral index contributed most to the spectral indices model using Landsat–8 OLI (Fig. 5). The FLH_violet and BDA2 spectral indices contributed the least to the overall performance of the model using Sentinel-2 MSI and Landsat-8 OLI data, respectively. However, the NDCI spectral index performed moderately when used with Landsat-8 OLI or Sentinel-2 MSI for the spectral indices model.

#### Spectral bands and indices model

**Fig. 6:**
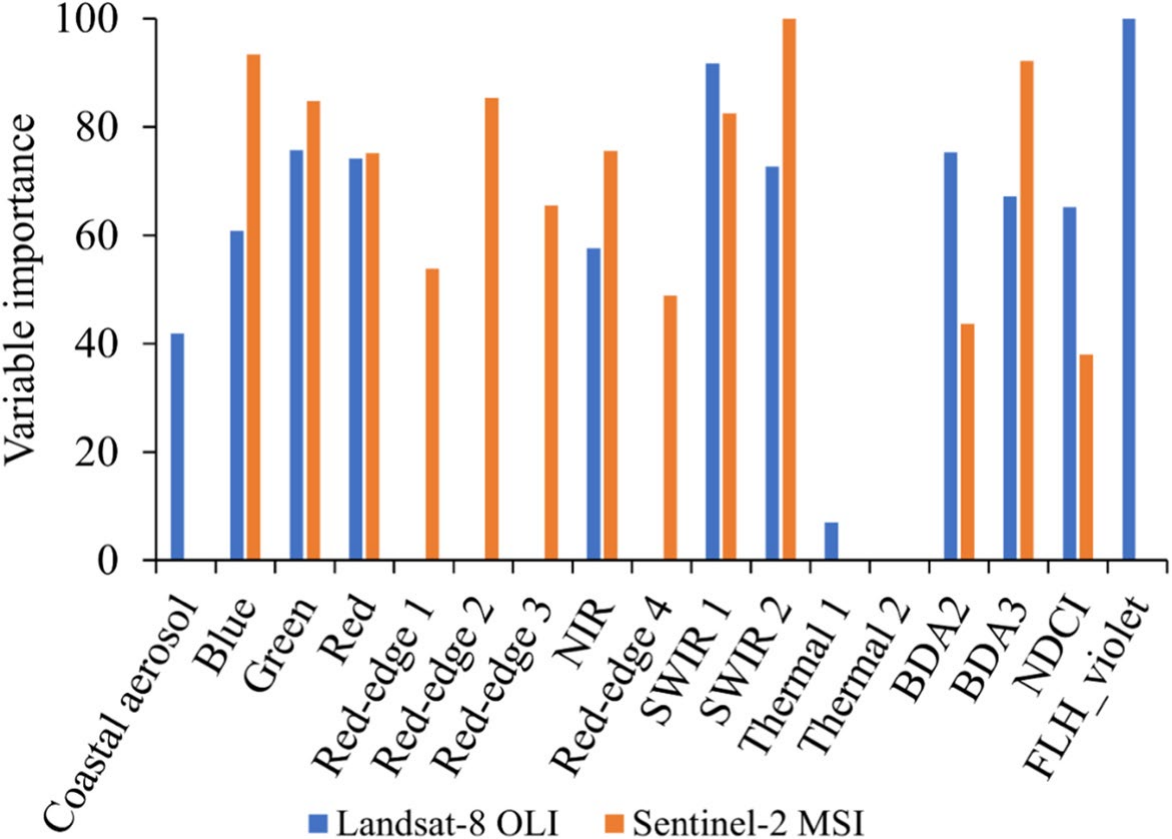
Variable contribution of Landsat-8 OLI or Sentinel-2 MSI derived spectral bands and indices to the performance of the spectral bands and indices model

The SWIR 2 spectral band, followed by the blues spectral band and the BDA3 spectral index, contributed most to the spectral bands and indices model using Sentinel-2 MSI data (Fig. 6). For the same model using Landsat-8 OLI data, the FLH-violet spectral index contributed the most, followed by the SWIR 1 and green spectral bands. The thermal spectral bands from Landsat-8 OLI and the FLH_violet spectral index from Sentinel-2 MSI contributed the least to the performance of the spectral bands and indices model when estimating chl-*a*.

## Discussion

Cost-efficient methods of monitoring eutrophication are essential for effective water management in aquatic environments, allowing for timely and accurate assessment of nutrient levels and the implementation of targeted mitigation strategies (Chawla et al., 2020; Sheffield et al., 2018). This study aimed to asses the water quality of Nandoni reservoir by comparing chl-*a* concentration using Landsat-8 OLI and Sentinel-2 MSI. The results are coherent with similar studies conducted in other environments and demonstrate how earth observation is valuable for monitoring water reservoirs in South Africa.

### Chlorophyl -a (Chl-a) variation across a spatial scale

The results showed that the concentration of chl-*a* was high at the edges of the Nandoni reservoir and closer to the reservoir wall when using Landsat-8 OLI and Sentinel-2 MSI data. The observed pattern in chl-*a* concentration can be attributed to physical and ecological factors influencing algal growth and nutrient availability (Moreno-Ostos et al., 2009). Consistent with previous studies, the accumulation of chl-*a* along the ends and near the reservoir wall can be attributed to reduced water circulation and increased sedimentation in these areas (Munyai et al., 2022). The restricted flow and hydrodynamic processes near the reservoir wall can accumulate nutrients and organic matter, creating favorable conditions for algal growth (Moreno-Ostos et al., 2009; Munyai et al., 2022). The localized hydrodynamic conditions create stagnation zones where algal biomass can accumulate and persist, resulting in higher chl-*a* concentrations (Pinardi et al., 2015). This phenomenon has been previously documented in studies focusing on factors influencing algal bloom distribution (e.g., Pinardi et al. (2015); Ahn et al. (2020); Chen et al. (2016)). On the other hand, the high concentration of chl-a near the edges could be explained by the spatial variations in light availability and penetration within the water column in shallow water (Depew et al., 2006; Rajendran et al., 2022). The edges of water reservoirs are often shallower compared to the central regions of the water body (Depew et al., 2006; García-Rodríguez & Tavera, 2002). As a result, these areas receive relatively higher amounts of incident light, allowing for greater photosynthetic activity and chl-*a* production (Rajendran et al., 2022). The increased light availability can stimulate algal growth and contribute to the higher chl-*a* concentrations observed in these regions (Cloern, 1999; Fork et al., 2020). These findings contribute to our understanding of the complex dynamics of chl-*a* distribution in reservoir reservoirs and have implications for water quality assessment and ecological monitoring in similar environments.

#### Sensor selection in estimation in chl-a concentration

The results from this study demonstrate that the model using spectral bands only from Sentinel-2 MSI had a high accuracy (OA = 0.87) when compared to the model using spectral bands only from Landsat-8 OL I (OA = 0.87). Landsat-8 OLI and Sentinel-2 MSI sensors have different spectral and spatial configurations that influence their use in estimating chl-*a*. Landsat-8 OLI provides data in the visible, near-infrared, and shortwave infrared regions, while Sentinel-2 MSI covers a broader spectral range, including additional bands in the red edge and atmospheric correction bands (Ngadze et al., 2020). The additional red-edge spectral bands in Sentinel-2 MSI have been crucial in observing the subtle difference in chlorophyll in most vegetated environments (Bramich et al., 2021; Zhang et al., 2022a, 2022b). In aquatic environments where chl-*a* exits, these bands have also been observed to be relevant (Bramich et al., 2021). Findings from this study are coherent with similar studies that have been done in Lake Erie (Bramich et al., 2021) and Beijing (Shi et al., 2022), where chl-*a* estimation was accurate when Sentinel-2 MSI spectral bands were used. In addition to the spectral configuration, the difference in spatial resolution between Sentinel-2 MSI and Landsat-8 OLI play a role when estimating chl-*a* since water’s absorption and scattering properties affect the chl-*a* concentration estimation (Dzurume et al., 2022).

The results also showed that the model using spectral indices, a combination of spectral indices and spectral bands, had a high accuracies when using Landsat-8 OLI data than when using Sentinel-2 MSI. However, the predictive accuracy of these models was not significantly different (*p* > 0.55). The difference in predictive performance between Sentinel-2 MSI and Landsat-8 OLI could be attributed to the choice of spectral indices utilized in the analysis. The selected indices, three-band algorithms (BDA), fluorescence line height (FLH_violet), and normalized difference chlorophyll index (NDCI), are known to be effective indicators of chl-a concentrations in various aquatic environments (Buma & Lee, 2020; Rajendran et al., 2022; Zhao et al., 2022). In this study, these indices performed better when calculated from Landsat-8 OLI. These findings are similar to the results by Buma and Lee (2020) and Karimi et al. (2022), who found out that these indices demonstrated a higher mean average when utilizing Landsat 8-OLI or yielded comparable outcomes to those obtained from Sentinel-2 MSI. The performance of these indices may also be attributed to the tailored nature of the FLH_violet index to the spectral characteristics of Landsat sensors (Buma & Lee, 2020; Johansen et al., 2018; Rajendran et al., 2022). As for the other indices (BDA and NCDI), it is plausible that they exhibit different sensitivities to chl-*a* concentration in diverse environments (Buma & Lee, 2020; Rajendran et al., 2022). Lastly, the contrasting accuracies may lie in the radiometric calibration of the data. Landsat’s rigorous calibration and validation processes, well-established over decades of satellite missions, could have contributed to the robustness of its derived indices (Nazeer & Nichol, 2014; Rajendran et al., 2022; Smith et al., 2021). On the other hand, although Sentinel-2 MSI also undergoes a rigorous calibration process, its comparatively recent operational deployment may result in subtle calibration differences, impacting the accuracy of the derived spectral indices (Tran et al., 2023).

### Limitations

While this study contributes valuable insights into chl-a concentration estimation using remote sensing, some limitations exist. The absence of multi-temporal in situ data for validation restricts our ability to comprehensively evaluate the accuracy of our remote sensing-based estimations. Multi-temporal data allows for assessing the consistency of the remote sensing-derived chl-a estimations across varying environmental conditions and seasons (Dzurume et al., 2022). Multi-temporal images could also improve the results since single images can be affected by atmospheric conditions and variations in water constituents can introduce uncertainties into remote sensing data, potentially affecting the precision of chl-a estimations (Kravitz et al., 2020; Rajendran et al., 2022). Furthermore, the research primarily focuses on specific sensors and spectral indices, which may limit its applicability in diverse environmental contexts. Future research should address these limitations by incorporating multitemporal in situ measurements and images and explore advanced techniques to enhance the reliability of chl-a concentration assessments. Nonetheless, the results from this study are relevant and have the potential of supporting water management efforts in South Africa.

### Implication on water management in South Africa

Several studies have emphasized the importance of chl-*a* estimation in reservoirs as a critical indicator of water quality and ecological health (Li et al., 2018; Watanabe et al., 2015). Elevated chl-*a* concentrations are often associated with eutrophication, which can lead to ecological and environmental issues (Li et al., 2018; Matthews, 2014; Nguyen et al., 2021). For instance, excessive algal growth and algal blooms can result in reduced water clarity, decreased dissolved oxygen levels, and alteration of the aquatic ecosystem dynamics (Nguyen et al., 2021; Pamula et al., 2023). Such conditions can impact fish populations, biodiversity, and overall water quality, necessitating targeted management interventions which might be costly (Dzurume et al., 2022; Matthews, 2014).

The comparison between Landsat-8 OLI and Sentinel-2 MSI for chl-*a* estimation in the Nandoni Reservoir provides valuable insights based on existing literature. Selecting suitable remote sensing data and sensors is crucial for accurate and reliable chl-*a* estimation (Buma & Lee, 2020; Dzurume et al., 2022). The choice of the sensor can affect the retrieval accuracy due to differences in spectral bands, spatial resolution, and atmospheric correction algorithms (Buma & Lee, 2020). Findings from this study align with studies that have highlighted the strengths of Landsat-8 OLI for chl-*a* estimation in diverse aquatic environments (Buma & Lee, 2020; Munyai et al., 2022). In addition, identifying higher chl-*a* concentrations along the ends and closer to the reservoir wall can inform management strategies to mitigate potential water quality issues. By recognizing these localized areas of high chl-*a* concentrations using earth observation technologies, water resource managers can implement measures to improve water circulation, reduce nutrient loads, and monitor and control algal blooms more effectively (Munyai et al., 2022). Lastly, the spatial distribution of chl-*a* concentrations derived from remote sensing data can also calibrate and validate water quality models, providing valuable information for future predictions and scenario analysis. These insights contribute to the knowledge of reservoir monitoring and assist in evidence-based decision-making for sustainable water resource management in the Nandoni Reservoir. Further collaboration between remote sensing experts and water resource managers is essential to translate these findings into effective and sustainable management practices for the Nandoni Reservoir and similar water bodies in South Africa.

## Conclusion

This study contributes to the broader understanding of using remote sensing as a tool for water quality assessment. The findings demonstrated that models using spectral bands from both Landsat-8 OLI and Sentinel-2 MSI performed comparably, highlighting the potential of both sensors for this purpose. Sentinel-2 MSI’s additional red-edge spectral bands provided a notable advantage in capturing subtle variations in chlorophyll levels, making it a promising option for future studies in various aquatic environments. These findings underscore the importance of carefully choosing suitable sensors based on specific environmental conditions when estimating chl-*a* concentrations using remote sensing data. To further advance this research area, future investigations should delve into exploring advanced data fusion, and machine learning techniques can help optimize chl-a estimation and promote more accurate water quality assessment using remote sensing technologies.

Insights from this study provide a foundation for improved water quality monitoring and management, and future research will refine our understanding and improve the reliability of satellite-based chl-a estimation.

## Data Availability

Data used in this research is freely available online and upon request.
